# How Resistance Shapes Health and Well-Being

**DOI:** 10.1007/s11673-022-10183-x

**Published:** 2022-04-06

**Authors:** Ryan Essex

**Affiliations:** The Institute for Lifecourse Development, University of Greenwich, London, England

**Keywords:** Resistance, Activism, Health, Healthcare, Power

## Abstract

Resistance involves a range of actions such as disobedience, insubordination, misbehaviour, agitation, advocacy, subversion, and opposition. Action that occurs both publicly, privately, and day-to-day in the delivery of care, in discourse and knowledge. In this article I will demonstrate how resistance plays an important (but often overlooked) role in shaping health and well-being, for better and worse. To show how it can be largely productive and protective, I will argue that resistance intersects with health in at least two ways. First, it acts as an important counterbalance to power; undermining harmful policies, disobeying unfair instructions, challenging rights abuses, confronting those who would otherwise turn a blind eye and even holding ourselves to account when simply accepting the status quo. Second, and beyond being oppositional, resistance is a constructive, productive force, that is fundamental to imagining alternatives; new and better futures and perhaps most fundamentally resistance is cause for hope that we are not resigned to the status quo. While there are numerous examples of how resistance has been employed in service of health and well-being, resistance is not always rational or productive, it can also harm health. I will briefly explore this point. Finally, I will offer some reflections on the intersections of power and health and why this makes resistance both distinct and important when it comes to how it shapes health and well-being.

## What is Resistance?

This paper is about resistance; more specifically to highlight its importance as it relates to health and well-being; as a positive force that has been and will continue to serve and protect health and well-being but also as a force that can be harmful to health. When I say “resistance” I mean it in its broadest sense. Resistance entails a range of actions; disobedience, insubordination, misbehaviour, agitation, advocacy, subversion, and opposition (Essex 2021). As it relates to health, resistance could be carried out by healthcare workers but also by virtually any individual or collective with some type of interest in health and well-being. Resistance includes action that occurs publicly but that also occurs day-to-day in the delivery of care. Perhaps more fundamentally I also refer to resistance that exists in discourse and knowledge; resistance that challenges assumptions, beliefs, and practices and what we think we know about certain things. The argument I present here is not to say we disobey for the sake of it, nor is it a case against obedience, why we should comply with authority or obey the law for example; resistance can be counter-productive, it can seek to maintain the status quo and entrench power; resistance can also be met with resistance. It is to say that too often we accept things as they are, too often we accept inequalities, unfair structures, broken systems, and the “truths” that perpetuate injustice, too often we accept those small indignities day-to-day that chip away at health and well-being, too often we fail to question authority, too often we fail to question ourselves. Equally, the role of resistance in securing and protecting health and well-being has largely been overlooked. In this article I will argue that resistance can serve as an important means to oppose threats to health, that is, structures, policy, laws, or the multitude of others factors that could negatively impact on health and well-being. Not only this, but resistance has a role to play in imagining alternatives to the status quo, that is, imagining how society could be better structured to support health and well-being or imagining alternatives to harmful systems and policy. In this article I want to fill this gap by drawing on two different conceptualizations of resistance, the first sees resistance as oppositional to but shaped by power, while the second sees resistance as more entangled with and inseparable to power. As a caveat I will also show how resistance can harm health. On the topic of what I am hoping to do, it is also worth briefly addressing what I am not going to do below. I do not want to dwell on the lack of definitional consensus or be caught up in discussions on resistance and its precise relationship to power for example. I also am not going to discuss distinct forms of resistance and their justification, such as civil disobedience; these conversations are of course important but are for another time. Before outlining my reasons in more detail let’s turn to a number of examples of resistance, to not only show how common such action has been as it relates to health and healthcare but to begin to sketch some further contours of what can be a relatively broad and contested concept (Vinthagen and Johansson 2013).

While we can trace “agitation”^1^ in public health back to the 1800s (and while I will say more about how many public health advances were fought for below; Paterson 1948) and while we can chart a history of similar action and agitation, we only need to look to recent history to illustrate the frequency and diversity of resistance. Throughout 2020, during the COVID-19 pandemic the “heroics” of healthcare workers were widely celebrated (Cox 2020), far less reported were the actions of those who opposed government responses to the pandemic, who demanded greater protections for themselves and their patients, who asserted that their “heroics” were only necessary because of decades of underfunding and neglect (Berger 2021). On the morning of September 15, 2020, a group of Ecuadorian medical students marched to the city centre in the capital, Quito, just outside the presidential palace demanding the government commit to a recently passed law that would guarantee them a contract and salary for the duration of the COVID-19 pandemic. Despite police using tear gas to disperse the students, this was only the first day of a four day national protest which included marches, sit-ins, and picketing. It was only after this strike that the government agreed to negotiate. By September 18, the government had agreed to meet the protesters demands and comply with the law that was introduced almost two months earlier (Ricci 2020). In neighbouring Peru at about the same time, protests by healthcare workers persisted throughout late 2020, demanding better working conditions and highlighting decades of under-investment in health (Associated Press 2021a). By early 2021, a memorial was set up for the 120 nurses and 260 doctors who had died because of COVID-19 (Associated Press 2021b). At the same time and again, not far away, on February 19, 2021, Bolivian healthcare workers began a strike, demanding the repeal of an emergency law which outlawed strikes and permitted the hiring of foreign health workers. In the days after the strike was called footage emerged of Bolivian riot police using tear gas on protesters (Reuters 2021).

Such action can be seen against the backdrop of broader unrest across the globe, with these events not only isolated to South America. In Myanmar, after the military coup in February 2021, healthcare workers had a central role in the broader protests across the country, initially going on strike, concerned about the state of democracy in the country, noting that they “simply [did] not want to work for the regime that staged the military coup” (Nachemson 2021, ¶4). As the situation deteriorated reports emerged that many healthcare workers had faced intimidation and harassment at the hands of security forces (World Medical Association 2021) and that by mid-February hundreds had gone into hiding as the government had sought their arrest (Htwe 2021).

As well as COVID-19, 2020 will also be remembered for protests against police brutality, particularly in relation to black and ethnic minority communities. Many of these protests began in the United States, where black Americans are far more likely than those of other ethnic backgrounds to be killed by police (Edwards, Lee, and Esposito 2019) and after a series of high-profile cases of brutality and police killings throughout 2020. These issues came to a head after the death of George Floyd. Floyd was an African American man killed during an arrest after a police officer knelt on his neck for almost nine minutes. Footage of Floyd’s death circulated globally, causing global protest; highlighting a public health issue, racism, equally as pressing (if not more so) than the COVID-19 pandemic. Health workers were again involved and joined part of a much larger outcry to address racial inequalities and police treatment of those from minority backgrounds. Dr Rhea Boyd, a paediatrician who joined the protests, noted that “protest is a profound public health intervention, because it allows us to finally address and end forms of inequality” (Ducharme 2020, ¶ 2). While almost all of the above examples focus on healthcare workers, they do not have a monopoly on such action as it relates to health, far from it. Over the last several years we can see hundreds of examples of the general public taking to street in making demands about their health, from vaccine mandates (Willsher 2021) to making demands about healthcare services (Ferguson 2021). More recently in the United States (BBC 2021) and Poland (Associated Press 2021c), thousands have taken to the street to protest strict abortion laws.

Even with these many examples, these largely public, mostly collective acts of resistance from healthcare workers, patients, and the general public only represent what is a small spectrum of action that could be considered resistance. Resistance also occurs day to day, it needn’t be public, collective, or even have demands attached. For example, we could resist by undermining structures of systems that harm health, care could be provided outside of traditional institutions for those who might not otherwise have access to healthcare. While this type of resistance rarely makes headlines, its impact can be just as significant as public and collective forms of protest. I will expand upon this point and offer some more examples below. My point should be made however, that resistance as it relates to health and well-being is remarkably common. Despite this however, the role of resistance in securing and protecting health and well-being has largely been overlooked. In this article I want to fill this gap by drawing on two different conceptualizations of resistance, the first sees resistance as oppositional to but shaped by power, framed by the work of Scott (1986). The second conceptualization sees resistance as more entangled and inseparable to power, framed by Foucault’s (2012) work on power and on more recent thought on resistance from authors such as Vinthagen and Johansson (2013) and Lilja (2021). Drawing on these conceptualizations I will show how resistance shapes health and well-being, acting as both a positive and negative force. I will first explore resistance as a means to respond to power; power that threatens, compromises, or impacts health and well-being; power that oppresses or coerces. Resistance can act as an important counterbalance to power; undermining harmful policies, disobeying unfair instructions, challenging rights abuses, confronting those who would otherwise turn a blind eye and even holding ourselves to account when simply accepting the status quo. My second point relates to resistance as being a constructive, productive force, as fundamental to critical thinking and in imagining alternatives. While resistance could be seen simply as a means to act in opposition and while in many cases this is somewhat true, this limits our inquiry; resistance is also fundamentally about imagining and pursuing something better. I will go on to offer somewhat of a caveat, showing how resistance could also be irrational, counter-productive, and even re-enforce existing power relations; ultimately resistance could also be harmful to health. Perhaps more generally and even with this caveat in mind, I hope to challenge common assumptions that obedience should be our default; we should be wary of the status quo, we should be sceptical of authority, and in some cases, we should take the streets in the name of health. The impact that resistance has on health and well-being is somewhat difficult to measure empirically, so I will illustrate my argument with a number of examples, both historical and more recent.

## Resistance as Opposition to Power

When we think about resistance, perhaps the first thing that comes to mind are the many examples of public protest discussed above. Such protests more often than not have relatively clear grievances or demands attached and often have a relatively clear opposition; the government, police, employers, or some type of policy or structure that may be seen as harmful. Resistance however also takes more subtle forms, occurring in day-to-day actions, where grievances might be less overt and opposition less well defined. On this, let’s turn to the concept of “everyday resistance.”

Everyday resistance is a concept that was introduced by Scott (1986) who contrasted it to open, organized resistance, such as marches or civil disobedience. Everyday resistance is less visible and often employed by groups who have relatively little power and thus luxury to openly confront their oppressors. In Scott’s (1989) words, everyday resistance is “virtually always a stratagem deployed by a weaker party in thwarting the claims of an institutional or class opponent who dominates the public exercise of power” (52). A number of further contrasts can be drawn with more open forms of resistance. Scott for example, argues that while objectives might be the same, compared to more open acts, everyday resistance “is ‘heavy’ on the instrumental side and ‘light’ on the symbolic confrontation side” (Scott 1989, 56). In practical terms everyday resistance includes action such as, “foot-dragging, dissimulation, false-compliance, pilfering, feigned ignorance, slander, arson, sabotage, and so forth” (Scott 1986). Such action is often also quiet, disguised and undeclared. Essentially, many everyday acts could be considered resistance. For those sceptical of this concept and that such action could be considered resistance, Scott argues that the form of resistance is largely shaped by the form of power in question, that is, those who claim that “‘real resistance’ is organized, principled, and has revolutionary implications … overlook entirely the vital role of power relations in constraining forms of resistance” (Scott 1989, 51). He goes on to note that if we only care for “real resistance” then “all that is being measured may be the level of repression that structures the available options” (Scott 1989, 51). With this understanding, resistance then not only opposes power via public, collective acts but day-to-day acts, sabotage, subversion, and even more subtle, individual acts. Below I will discuss some examples of how this could apply to health.

In 1942, Nazi Germany occupied Poland. Millions died during the occupation and many groups, such as the Jews, were singled out, persecuted, and sent to their deaths (United States Holocaust Memorial Museum 2020). After World War I the Nazis were particularly weary of communicable diseases, having been exposed to a number of outbreaks during the war. German authorities required authorities in Poland to report all suspected communicable diseases, including Typhus. Polish nationals diagnosed with the disease were spared being sent to labour camps and were quarantined. Jews with the disease were executed (Chapoutot 2014). Eugene Lazowski, a doctor who already had a long history of subversion and defiance recognized this and instead created what could best be labelled a fake Typhus epidemic. Lazowski and his colleagues, after learning how to administer the test for typhus, recognized they could create a false positive result, by injecting patients with dead antibodies. This “epidemic” was soon large enough to declare a number of villages an “epidemic area,” which meant the withdrawal of German troops allowing the population to live with relative freedom (Bennett and Tyszczuk 1990). When the German authorities questioned the low mortality rate due to the epidemic, a committee was assembled to investigate. When meeting German officials, Lazowski and is colleagues threw a lavish party, while also assembling the most unwell-looking people in the village and having them huddle in filthy huts. After a cursory inspection, with more senior members occupied by the reception, the German committee left and did not return. All of this occurred in secret and was only disclosed in the 1970s, with the doctors not even revealing what they were doing to their patients. It was estimated that the doctors saved an estimated eight thousand people from being killed or imprisoned during their three-year campaign (Goor 2013).

Two more recent examples, shed more light onto how resistance could serve health. In 2011 many residents in Ghouta, a small suburban area on the outskirts of Damascus joined protests against the Syrian government. Amid a government siege, Dr Amani Ballour abandoned her paediatric training to begin to treat those impacted by the violence, volunteering at a hospital held by rebel forces (Ballour, in Bseiso, Hofman, and Whittall 2021). At the same time, a few hours north in Aleppo, Dr Hamza al-Kateab formed a hospital to begin to treat those again impacted by the violence in Aleppo (Prasad 2019). Both continued to work despite the risks they faced; violence, frequent attacks by the government and the death of hospital staff. In late 2016, as the government re-took Aleppo, al-Kateab evacuated the hospital while in Ghouta, Ballour was elected and promoted as the hospital director; the first and only female in Syria to hold this position. She ran the hospital until the government quelled the last resistance in 2018, after which she was forced to flee to a refugee camp in Turkey (Willsher 2020). Both Ballour’s and al-Kateab’s stories are captured in the films “The Cave” (Fayyad 2019) and “For Sama” (Al-Kateab and Watts 2019) respectively. These films, that document the atrocities committed in Syria, were acts of resistance in themselves, as was the act of staying and providing care. Most fundamentally, the Syrian government’s intent was to make life as unbearable as possible for its own citizens, to starve people out, to re-take territory by attrition. Staying was an act of resistance, providing healthcare was an act of resistance. This was not lost on Waad al-Kateab, the director of For Sama, who characterized the uprising in Syria as a “revolution against oppression” and noted that if put in the same situation again, many would “still resist until their last breath” (Kanawati 2020, ¶8).

Beyond the dramatic examples above, resistance occurs in far less exceptional circumstances. In a study that examined everyday resistance in how medical students responded to the professional lapses of more senior staff, Shaw et al. (2018) found that everyday resistance amongst medical students was a frequent occurrence and took a multitude of forms, including verbal, bodily, and psychological forms of resistance. Furthermore this research highlighted the often subtle and nuanced ways in which resistance was acted out, in this case in acts that challenged or undermined professionalism lapses of more senior clinicians. Simple acts such as closing curtains for privacy when others had left them open or verbally challenging unprofessional behaviour were common, challenging discourse that was unprofessional or modelling generally accepted professional behaviour. In this case, the authors noted that resistance served several important purposes in immediate practice, from alleviating distress, to addressing lapses in care, and in raising awareness about inappropriate actions, resulting in behaviour change. Beyond these more immediate impacts, resistance served to more subtly challenge dominant and taken for granted structures; undermining hierarchy for example.

From occupied Poland to otherwise unremarkable hospital wards, resistance has been and will continue to be utilized by healthcare workers and the healthcare community as a means to protect health and resist oppression. Resistance could involve open, collective acts, such as protest marches or civil disobedience. It could also occur in various everyday acts, such as those documented above in occupied Poland and Syria. Such forms of resistance could involve disobeying an order, it could mean subverting or undermining harmful policy that in some way harms health or well-being. The action of Dr Amani Ballour and Dr Hamza al-Kateab in simply staying and providing care throughout the Syrian civil war were acts of resistance. In the above examples we can see how resistance is shaped by the form of power to which it is opposed. Under most circumstances, staying and doing your job would not be an act of resistance. Under most circumstances, injecting patients without their consent would be unconscionable, in Nazi occupied Poland such action became an act of resistance and saved thousands of lives. The above examples show how resistance has been used to challenge, undermine, and ultimately counter power that threatens health. Even though the above conceptualization of resistance could involve any range of activities, such an understanding of resistance is still somewhat of an oversimplification. While resistance is fundamentally oppositional, this conceptualization risks reducing it to something that is reactionary; it is more than this, resistance is entangled with power. This leads to my second point that resistance can also be a constructive, productive force.

## Resistance as Constructive

Few would argue that resistance is most fundamentally oppositional, it is a concept that can only exist in opposition to something else. To understand how resistance serves as a constructive force, we need to reconceptualize how we understand resistance and to do this we first need to understand how power is conceptualized. On this point, we first turn to Foucault. While Foucault spent comparatively little time speaking about resistance, his ideas about power are particularly important here. For Foucault (2012), power was not wielded, it was ubiquitous, diffuse, and without individual authors. Power was essentially everywhere and was embodied in knowledge and “regimes of truth” (Foucault 2012). Foucault described regimes of truth as discourse that society

… accepts and makes function as true; the mechanisms and instances which enable one to distinguish true and false statements, the means by which each is sanctioned; the techniques and procedures accorded value in the acquisition of truth; the status of those who are charged with saying what counts as true. (Foucault 1980, 131).

In other words, power dictates the way any topic can be meaningfully discussed. Building on this conceptualization of power, many have reconceptualized resistance as something that is not just oppositional but something that is far more complex and entangled with power.

Vinthagen and Johansson (2013) for example, argue that as resistance exists in relation to power, this also means that resistance can also be ubiquitous, diffuse, and without individual authors. They go on to argue that this essentially means that power and resistance are interdependent which makes it almost impossible to separate them, instead leaving us with what they describe as an entanglement. This also means that we cannot understand resistance without first understanding the power to which it is entangled. While this adds further complexity to this picture it also opens up a range of possibilities for resistance, what it is and how it works, beyond simply existing as an oppositional force. Returning again to the ideas above, if power is diffuse and without authors, if power exists in knowledge and truths we otherwise take for granted, one of the most fundamental functions of resistance is to imagine alternatives, overcoming these assumptions and creating new possibilities. For Hayward and Schuilenburg (2014) resistance serves as a “solvent of doxa, to continuously question obviousness and common sense, in order to create a new image of thought, and thus to remind us that things do not have to stay the way they are” (34). For Flohr (2016) resistance “occupies a threshold between contemporary configurations of power and the possibility that things might be otherwise” (50). In developing this perspective Lilja (2021) has made a substantial contribution essentially arguing that through repetition and over time, resistance can (and has) “produce[d] new and emerging realities,” new ways of thinking, new ways of viewing old problems while promoting a different and better vision for the future. This may have already been evident in some of the examples above, but let’s again consider some further examples below.

In England in the 1840s most would not be expected to live beyond forty years old (Office for National Statistics 2015). For the working class the outlook was even more bleak with the squalid conditions in which they lived and worked described as “scandalous” (Engels 1993). The Health of Towns Association was formed in 1844, with the aim of educating the public and pushing for public health legislation. The association pushed for this change utilizing a number of means. A major aim of the association was educational, that is, it sought to educate people on sanitation, producing a number of reports. Much of its action however was also agitational and inspirational, making “urban sanitation the crusade of the day” (Hamlin 2008, ¶6). The association also organized petition drives and meetings, highlighting the failures of the government and presenting new visions for the “sanitary kingdom to come” (Hamlin 2008, ¶6). The Public Health Act was passed in 1848 and having achieved this major objective, soon after the association dissolved. While it now seems somewhat obvious that public action could improve population health, this was a controversial idea in the 1840s. At this time in Europe for the working classes in particular, sanitation was near non-existent and more often than not government responded to epidemics with decrees, “set their military forces to protecting borders and ports, whitewashed towns, fumigated dwellings, and burnt bedding” (Hamlin and Sidley 1998, 587). The Health of Towns Association was not only remarkably successful in “creating a will to act” (Hamlin and Sidley 1998) but in also articulating a new and better approach to public health. While the Health of Towns Association employed a range of action that sought change, some of it oppositional and agitational, a substantial part of this work involved simply promoting the idea that an alternative, a better approach to sanitation was possible. Substantial effort was put into “inspirational” efforts promoting this idea amongst the working class, not only drawing them in to existing advocacy efforts but in having them imagine better for themselves and their communities.

Let’s consider one final example that perhaps gets to the heart of Foucault’s ideas about power and “truth.” In 1973, the American Psychiatric Association (APA), included a symposium at its annual meeting that debated whether homosexuality should exist in the APA’s nomenclature. At the time, homosexuality was pathologized and considered a psychiatric disorder (Stoller, et al. 1973). In the years leading up to this decision activists engaged in a number of actions to raise awareness about the stigmatization of this “diagnosis” but to also ultimately call for its abolition as a diagnosis. While several factors led the APA to remove homosexuality from the DSM, this resistance played a large part in securing this change. While there was still much to be done beyond this point, this change signalled the beginning of the end of American psychiatry’s stigmatization of homosexuality, with similar shifts occurring internationally over the next few decades (Drescher 2015). While significant, these changes came about within a larger shift in society, that challenged discourse in relation to homosexuality. As Foucault notes, what emerged was a form of “reverse discourse” where “homosexuality began to speak in its own behalf, to demand that its legitimacy or ‘naturality’ be acknowledged, often in the same vocabulary, using the same categories by which it was medically disqualified” (Foucault 1990, 101). Drescher (2015) argues the APAs decision meant that “religious, governmental, military, media, and educational institutions were deprived of medical or scientific rationalization for discrimination” (572) and furthermore, this shift changed health discourse in relation to homosexuality from questions such as “how can it be treated” to questions of how to support the needs of LGBTI patients (Drescher 2015). Perhaps most fundamentally this example shows how accepted “truths” dictated how homosexuality was viewed. Without challenges to the legitimacy of these “truths” it is likely that homosexuality would have remained pathologized beyond the 1970s and persisted as a legitimate means to discriminate.

Again, while I have focused on two examples above, all of which made substantive contributions to health and well-being, there are many more. Outside of these more specific examples we also only need to look at approaches toward disability, gender, and race in medicine to see such shifts. Stramondo for example argues that acts such as civil disobedience can be effective in counter-narrative to common disability narratives of pity and suffering. He points out that it is

… very difficult to pity someone who is deliberately using their 300lb power wheelchair to block your commute to the office. You might be angered by them or you might even admire them, but you almost surely will not pity them … (Stramondo 2021,12)

In all of these cases, resistance opened up a range of new possibilities; challenging how sexuality was pathologized and pushing for new approaches to public health. Change would not have occurred without actions that articulated alternatives, pointed out where we were wrong or complacent, challenged the status quo, and inspired others to think beyond the truths we too often take for granted. Most fundamentally, resistance is fundamental to critical thinking, without it there would be acceptance, obedience, little reason to move beyond the status quo. Looking at these historical examples there is a further point here: that medical history is littered with failures and complacency in the face of atrocities. We should be cautious not to repeat these mistakes but also not to sit idly by, we should resist.

## Resistance that Harms Health and Well-being

I am sympathetic to resistance and its potential to positively impact health. I do hope that anyone who reads this can see its value in promoting and protecting health and at least to some extent agrees that in many cases we should consider it as an important means to protect health. This however is not the entire story. Resistance cuts both ways, as it is entangled with power. And as power is not only oppressive but productive, it follows that resistance could also be irrational, counter-productive, and even re-enforce existing power relations.

Even from the examples above, we can see that healthcare workers for example can be both resistors and oppressors, whether in the study by Shaw et. al. or when looking at the pathologization of homosexuality in the 1970s. Beyond this however, resistance can also be met with resistance. While the American Psychiatric Association eventually shifted its position, resistance against pathologizing homosexuality was also met by resistance from the medical establishment and those within the American Psychiatric Association. This shows how resistance may be entangled with power but also how it may be used not only for positive change but also as a means to maintain the status quo and oppression. While far less reported in the literature, we can see other examples. In 1934 Montreal interns went on strike because Montreal’s Hôpital Notre-Dame sought to hire a Jewish doctor (Halperin 2021). More recently Indian doctors have protested a quota system to promote those from “lower castes” being admitted to medical colleges (Chatterjee 2006). Perhaps one final, more clear-cut example, comes from recent history.

A recent event that will be long remembered was the 2021 capital riot in the United States. On January 6, the United States Capitol was stormed by a mob of supporters of President Donald Trump. Amongst those was Dr Simone Gold. About six months earlier, Gold gained public attention when she and other physicians appeared in front of the Supreme Court for a sparsely attended news conference to decry pandemic lockdowns and criticize government efforts to stop the spread of the disease. Video of the event, organized by conservative activists, was retweeted by the president and viewed by millions before social media platforms took it down (Satija 2021). Gold was also founder of “America’s Frontline Doctors,” an organization that attacked policies advocated by mainstream scientists and healthcare workers, while promoting misinformation at rallies and on right wing news outlets. It remains to be seen the impact of this action, however throughout the COVID-19 pandemic, there have been multiple and ongoing protests against lockdowns and measures such as mandated mask wearing and, more recently, mandated vaccination.

## The Intersection of Resistance, Power, and Health

I have argued that resistance has and will continue to make a positive contribution to health and well-being, both by offering an opposition to power that would otherwise harm health and well-being but also by challenging “truths” and promoting different ways of thinking and articulating new and better futures. While some caution is warranted, we should not make obedience our default. While the reasons we should obey may seem self-evident, there is also an equally important case for resistance; it too can serve to protect and promote health and well-being.

For those familiar with the literature on resistance these points may not be new; resistance can be conceptualized as both a response to power and entangled with power. Beyond what I have said above I want to offer some further reflections on the intersections of power and health and why this makes resistance both distinct and important. These points are not exhaustive but should be seen as a starting point for future discussions in this area. First, health provides a powerful platform on which resistance can be framed. This arguably comes back to many seeing health as a particularly special good. That is, health can be seen as a precursor to being able to function as an agent and enjoy other freedoms and opportunities (Anand 2002). From the examples above and elsewhere we see many protests for many different reasons framed in terms of health; climate change, the detention of migrants, nuclear disarmament, and even anti-vaccine protests in response to COVID-19 have all been framed in terms of health. We can see this elsewhere, patient health and well-being is almost always cited as a grievance when healthcare workers pursue strike action. On this point, there is evidence to suggest that framing these issues in terms of health can have positive effects. For example, several studies have suggested that framing climate change as a public health issue is effective and could engage those previously not engaged and raise support for climate change mitigation and adaptation (Myers et al. 2012; Maibach et al. 2010). In many circumstances health has been used to give issues legitimacy and in garnering support; it can serve as a powerful way to frame resistance under the right circumstances. A second related point, is how resistance is shaped by the asymmetry of knowledge related to health and well-being and how this may lend further legitimacy to resistance. In visible forms of resistance healthcare workers often openly identify as such. Signs, scrubs, white coats, and stethoscopes are all examples. Throughout the 2019 Doctors for Extinction Rebellion actions in the United Kingdom a number chose to wear their scrubs “thinking that the media would find it harder to dismiss medical professional protestors as cranks” (Fulchand 2019, 1). Similarly, in response to nuclear disarmament Young (2019) argued that

… [t]aking a medical standpoint against nuclear weapons became a problem for the government, who could easily write off movements like the CND [Campaign for Nuclear Disarmament] as unwashed hippies … but when you have a doctor saying the same thing and backing it up with a medical argument, it’s much more difficult for the government to discredit. (e222)

Beyond these examples here, we can see above how the authority of healthcare workers has also been used to oppress, like the above argument, how health-related knowledge and authority cuts both ways. Beyond simply being entangled with power, resistance intersects with health in numerous ways, shifting the sites and nature of power and resistance. Like many other questions related to resistance and health, there is scope for further discussion on this point, along with discussion on the many unanswered questions about the normative aspects of resistance. Even from the examples above, we can see how forms of action, their objectives (or lack thereof), the context in which they occurred, the power to which they were entangled, and the risks that came with resisting all varied substantially. While there has been some work exploring the normative aspects of specific episodes or acts of resistance (Essex and Weldon, In press) or opposition that occurs in relation to specific issues, such as climate change (Bennett et al. 2020) or immigration detention (Essex 2020), less has been said about resistance more generally.

This is of course not the final word nor is it the whole story. The above discussion should not be seen as exhaustive or as the only intersections of health and resistance; nor should it be seen to diminish other important struggles. One example, which I have not discussed, relates to resistance by Indigenous populations. The ongoing impact of colonization and imperialism has had a devastating impact on the health and well-being of Indigenous populations with dramatic iniquity that continues to this day (Kauanui 2016). In Australia, for example, Indigenous life expectancy remains at least ten years less than the national average (Zhao et al. 2013). Resistance in this context is not only entangled with health and power but could be seen to serve an existential function, as a means of survival, a means of self-determination. The ongoing resistance of Indigenous populations is not only “testimony to ongoing dispossession” (Picq 2017, 2), but more profoundly indigenous survival “means that the project of the nation-state did not triumph … that there is not one single territory, not one single language, not one single citizenship” (Gladys Tzul in Capiberibe and Bonilla 2015, 293). For Palmater (2019, 131) “the most radical thing that a person can do … is be born Indigenous.” It is unfortunately beyond the scope of this paper, but it should go without saying that the intersections of health and indigenous resistance deserve far greater attention that what I have offered here.

Putting these things aside, and despite the need for future discussion, I hope my overarching point remains—resistance has been a common but overlooked force in health and healthcare that has been central to securing a number of health-related gains. I have argued that resistance can serve as an important means to oppose threats to health and that resistance has a role to play in imagining alternatives to the status quo, that is, imagining how society could be better structured to support health and well-being or imagining alternatives to harmful systems and policy. In short, I hope to have shown how resistance has been and will continue to serve as a positive force to hold power to account but to also inspire new and better futures. While not an explicit aim of this paper, I also hope I have shown how common and diverse such action can be and why it deserves greater attention. Even with the above caveats about the potential misuses of resistance in mind, looking to history, without resistance we simply would not have made many advances that we today take for granted. Resistance will continue to be a frequent occurrence as it relates to health and healthcare, from day-to-day action to marches, protest, and civil disobedience, there is substantial scope to discuss its conceptual and normative potential.

## Footnotes

1 Although this isn’t elaborated on specifically, this appears to be meant in a very general way. Action undertaken by the Health of Towns Association included marches, petitions, meetings, and publications that functioned as “inspirational” or calls to action (Hamlin 2008).
